# Using Capillary Whole Blood to Quantitatively Measure Ferritin: A Validation Trial of a Point-of-Care System

**DOI:** 10.3390/nu15061305

**Published:** 2023-03-07

**Authors:** Joanna L. Fiddler, Michael I. McBurney, Jere D. Haas

**Affiliations:** 1 Division of Nutritional Sciences, Cornell University, Ithaca, NY 14853, USA; 2 Department of Food, Nutrition, and Packaging Sciences, Clemson University, Clemson, SC 29634, USA; 3 Department of Human Health & Nutritional Sciences, University of Guelph, Guelph, ON N1G 2W1, Canada; 4 Friedman School of Nutrition Policy and Science, Tufts University, Boston, MA 02111, USA

**Keywords:** ferritin, diagnostic device, finger stick, blood, capillary, venous, nutritional deficiency

## Abstract

Iron deficiency is a public health problem with devastating health, developmental and behavioral effects which often exacerbated due to affordability and access to screening and diagnosis. Using *IronScan*™ a portable, point-of-care diagnostic system capable of quantitatively measuring ferritin in blood, we validated *IronScan™* ferritin measurements using whole blood and serum with a lab-based, regulator-approved analytical device for measuring ferritin in venous serum. Capillary (finger stick) and venous whole blood samples were obtained from 44 male and female volunteers. Venous serum (vSer) ferritin concentrations were measured on Immulite 2000 Xpi (gold standard). Capillary whole blood (cWB), venous whole blood (vWB), and vSer ferritin levels were measured by *IronScan™*. cWB ferritin concentrations from *IronScan™* were significantly correlated (R^2^ = 0.86) with vSer measured with the FDA-approved Immulite system. The results from the multiple regression analysis indicate that 10% of the variability was due to the method of blood collection (venous vs. capillary) and 6% was due to the form of blood analysis (whole blood vs. serum). The sensitivity of diagnosing iron deficiency using the WHO cutoff of <30 ng/mL is 90%, with a specificity of 96%. In conclusion, *IronScan™* is a rapid viable option for measuring ferritin as a point-of-care system.

## 1. Introduction

Iron deficiency is the most common cause of anemia. Anemia is characterized by a reduced number of red blood cells often accompanied by diminished hemoglobin levels or altered RBC morphology, and is associated with increased risks of maternal and child mortality and reduced cognitive and physical performance [1,2,3,4,5]. In 2010, anemia accounted for 68.4 million years of life lived with disability (YLD) globally [6]. The Global Burden of Diseases, Injuries, and Risk Factors Study (GBD) quantified incidence, prevalence, and YLDs for 354 non-fatal health outcomes, 22 Level 2 and 167 Level 3 causes. Anemia was the leading impairment of YLD (for both sexes) and ranked as the 4th leading Level 2 and the leading Level 3 cause in terms of age-standardized YLD rates globally for females in 2017 [7]. The etiology of anemia is multifaceted; recent meta-analyses estimate that 50% of anemia cases can be attributed to iron deficiency [8]. Therefore, accurate determination of iron status is crucial for diagnostic and screening purposes in clinical settings and to guide public health interventions at the population level [9]. The WHO recommends blood ferritin concentration as a good marker of iron stores in apparently healthy individuals and in populations, with adjustment for α-1 acid glycoprotein (AGP) and/or C-reactive protein (CRP) for individuals with infection or inflammation [9]. Using validated biomarkers such as blood ferritin to objectively assess an individual’s iron status, it is possible to personalize dietary guidance and management of those diagnosed with iron deficiency. As an example, screening the ferritin levels of blood donors and informing those who had low ferritin results led donors to see a health care provider, increase their iron consumption, and return to donate 6 months later when their iron stores were normal; this resulted in a modest increase in the ferritin levels of returning donors [10]. Females at high risk, e.g., during menstruation, pregnancy, and lactation [11], can be tested to confirm that their iron status is optimal or being normalized by following therapeutic instruction. At the population level, the implementation of representative sampling is a promising strategy to identify and monitor groups at risk, e.g., iron deficiency in young children (1–3 years) [12,13]. The use of validated nutritional biomarkers, e.g., blood ferritin, can provide an objective assessment of the iron status of populations to guide resource prioritization and public health action, whether it be nutritional interventions or regulatory action. Finally, ongoing representative surveys are the best way to assess the efficacy of public health actions.

The majority of locations in which ID is prevalent do not have access to or the clinical capability to measure these biomarkers [14]; therefore, a portable, point-of-care, diagnostic device using capillary whole blood to measure ferritin could facilitate screening, diagnosis, and the implementation and monitoring of public health interventions. The methodology employed in this report is similar to other lateral flow immunoassay-based point-of-care diagnostic systems for quantifying human analyte concentrations [15,16]. This paper validates the performance of *IronScan™*, a proprietary point-of-care lateral flow assay designed to measure ferritin in capillary whole blood that was provided by VitaMe Technologies, Inc. (doing business as VitaScan). 

## 2. Materials and Methods

**Figure 1:**
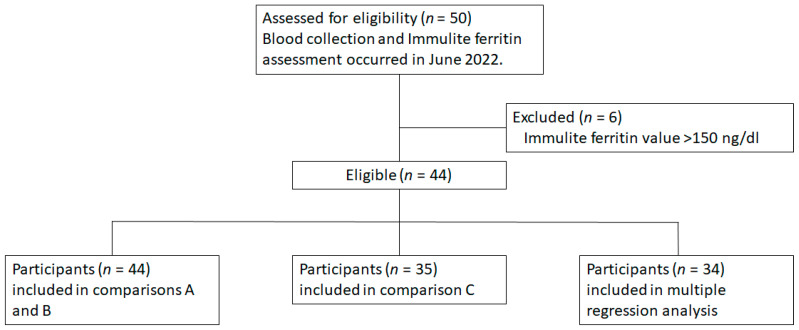
CONSORT diagram showing the inclusion and exclusion criteria and the resulting eligible participants, whose blood samples were further subdivided and analysed based on blood collection methods obtained during the data collection periods.

*IronScan™* validation was completed using different blood samples (whole blood, serum) and at different sample sites (capillary, venous) with venous serum (vSer) measurements using a lab-based Immulite 2000 Xpi analyzer. VitaScan™ (Ithaca, NY, USA) provided calibrated cube readers (*n* = 6) manufactured by ChemBio (Berlin, Germany) and lateral flow immunoassay cartridges to measure ferritin in a proprietary system, i.e., *IronScan*™. The validation trial was conducted 27–29 June 2022 in the Human Metabolic Research Unit of the Division of Nutritional Sciences at Cornell University after obtaining approval from the Cornell University Institutional Review Board. Forty-four volunteers (37 female, 7 male) were recruited from a convenience sample of students, faculty, staff, and the community of Ithaca via an email advertisement (Figure 1). All participants provided written informed consent and completed a health history questionnaire prior to participation. Individuals < 18-year-old were excluded from the study. A trained phlebotomist collected finger stick whole blood (cWB) and venous whole blood (vWB) by venipuncture (~5 mL). cWB and vWB were immediately analyzed by *IronScan™*. vWB was centrifuged to obtain serum (vSer) and was analyzed by Immulite 2000 Xpi (Siemens Medical Solutions USA, Inc., Malvern, PA, USA) and *IronScan™* on the collection day. All volunteers were nominally healthy. Two subjects were anemic based on hemoglobin (Hb) analysis from a Beckman Coulter Counter (Hb < 12.0 g/dL).

The objective of the study was to compare a portable, point-of-care, diagnostic system (*IronScan™*) with a lab-based, regulator-approved analytical device for the measurement of blood ferritin in venous serum, i.e., a gold standard system, Immulite 2000 Xpi [17]. *IronScan™* was designed to identify iron deficiency with highest sensitivity at low- to mid-range ferritin values; therefore, samples with Immulite ferritin values > 150 ng/mL were excluded from analyses (Figure 1). All *IronScan™* measurements were conducted in the Human Metabolic Research Unit, and all Immulite measurements were conducted in the Nutritional Chemistry Laboratory of the Division of Nutritional Sciences at Cornell University.

Three research questions were to be answered:

A. How do the diagnostic devices compare under expected usage?
  - *IronScan™* cWB blood vs. gold standard vSer in Immulite

B. How do the diagnostic devices compare when using the same collection site (venous)?
  - *IronScan™* vWB whole blood vs. gold standard vSer in Immulite

C. How do the diagnostic devices compare when using the same collection site and form of blood (vSer)?
  - *IronScan™* vSer vs. gold standard vSer in Immulite

All data analyses were completed with JMP^®^ Pro statistical software version 15 (SAS Institute Inc., Cary, NC, USA) or Graphpad Prism 9.5.0. Correlation analysis, R^2^ values, and a Bland–Altman plot were calculated to compare the performance of the *IronScan™* with the laboratory standard measurements made with the Immulite 2000 Xpi. Multiple regression analysis was used to compare all *IronScan™* comparisons to the Immulite 2000 Xpi. Sensitivity and specificity analyses were conducted using the WHO cutoff for ferritin (<30 ng/dL) and the clinical cutoff for ferritin (<12 ng/dL), using methods previously described and adapted for our study [18,19]. Briefly, sensitivity is the probability that the *IronScan™* system will diagnose iron deficiency among those with iron deficiency as measured by the Immulite 2000 Xpi (true positives). Specificity is the fraction of those without iron deficiency (true negatives as measured by the Immulite 2000 Xpi) that test as non-iron deficient on the *IronScan™* system.

## 3. Results

### 3.1. Subject Characteristics

The subjects were healthy male and female adults (Table 1). The results of the analysis follow the order of the questions presented above.

#### 3.1.1. Comparison A: Diagnostic Device Comparison under Expected Usage

**Figure 2:**
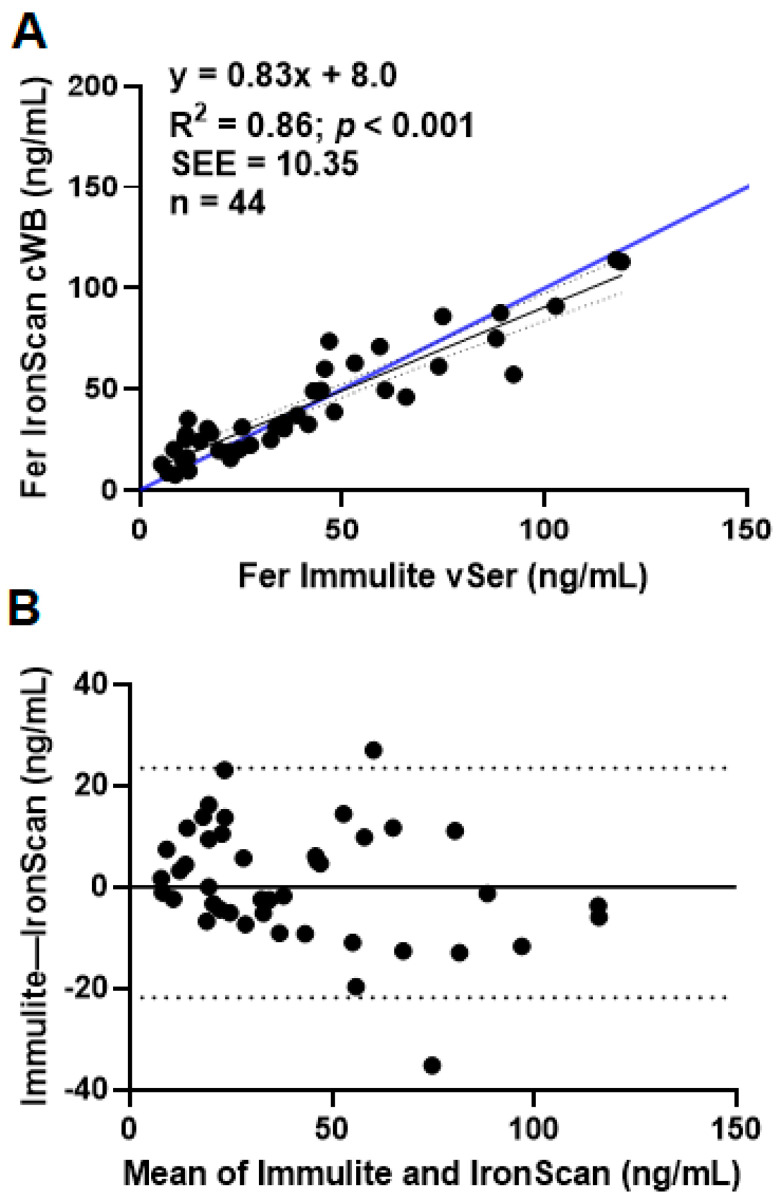
Comparison of ferritin (Fer) values from *IronScan™* using capillary whole blood (cWB) versus Immulite 2000 Xpi using venous serum (vSer). (**A**). Regression analyses. (**B**). Bland–Altman plot. Dashed lines represent 95% confidence intervals around the slope (panel **A**) or the mean (panel **B**). Blue line represents the line of identity. Standard error of the estimate, SEE.

Mean ferritin values analyzed across devices and blood sampling methods (Table 2) were similar for the primary comparisons (Immulite vSer versus *IronScan™* cWB). Ferritin measured by *IronScan™* in venous whole blood (vWB) is 38% higher than the mean values from capillary whole blood (cWB; the differences were not statistically significant by paired *t*-test (*p* = 0.66)). Ferritin cWB ferritin concentrations measured by *IronScan™* were significantly correlated (R^2^ = 0.86) with the Immulite system vSer ferritin concentrations (Figure 2A). A Bland–Altman analysis revealed only two values fell outside the 95% confidence interval (Figure 2B), and the greatest deviation for the regression is for the values above 60 ng/mL.

#### 3.1.2. Comparison B: *IronScan*™ versus Immulite Using Whole Blood or Serum from the Same Venous Collection Site

**Figure 3:**
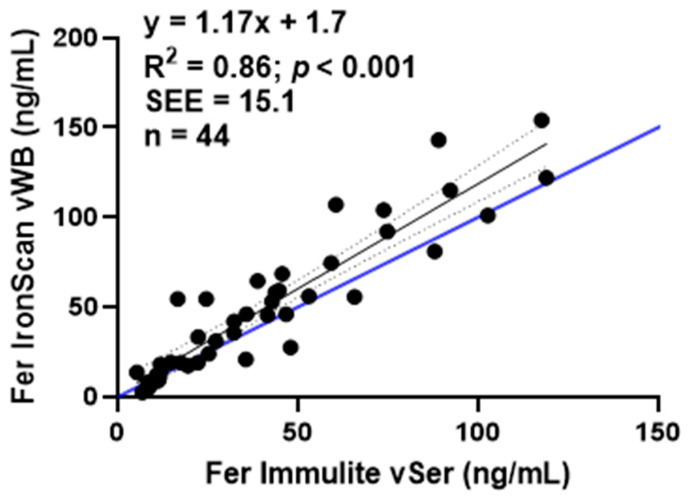
Comparison of ferritin (Fer) values from *IronScan™* using venous whole blood (vWB) versus Immulite 2000 Xpi using venous serum (vSer). Dashed lines represent 95% confidence intervals around the slope. Blue line represents the line of identity. Standard error of the estimate, SEE.

To determine whether sampling blood from capillary versus venous collection sites results in bias from the previous analysis (Figure 2), we used venous blood to determine ferritin values from both the Immulite and *IronScan*™. Ferritin values measured in vWB or vSer samples by *IronScan*™ and Immulite, respectively, were highly and linearly correlated (R^2^ = 0.86, Figure 3). The reduced slope is attributed to the *IronScan*™ overestimation of ferritin at higher values (<60 ng/mL).

#### 3.1.3. Comparison C: *IronScan*™ versus Immulite Using Serum Obtained from the Same Venous Collection Site

**Figure 4:**
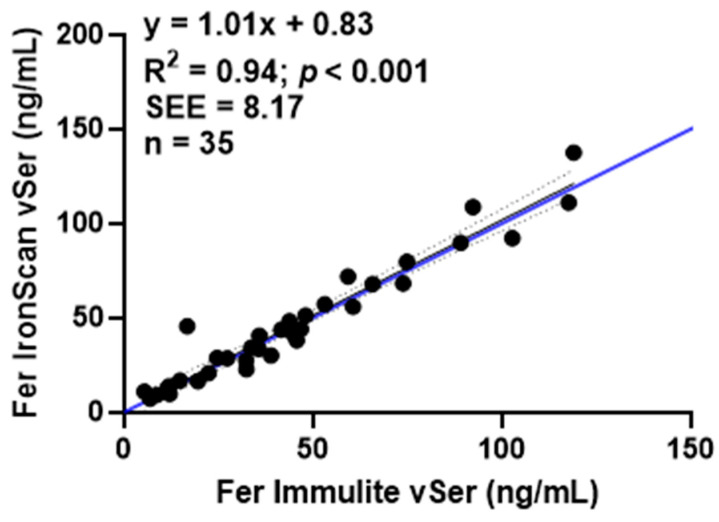
Comparison of ferritin (Fer) values from venous serum (vSer) samples using *IronScan™* versus Immulite 2000 Xpi. Dashed lines represent 95% confidence intervals around the slope. Blue line represents the line of identity. Standard error of the estimate, SEE.

To test the effect of the medium of blood used in the original analysis (Figure 2), we compared the Immulite versus *IronScan™* using only serum obtained from a blood venous sample for a subset of 35 subjects (Figure 1). When ferritin concentrations were measured for both devices using the same sample site and medium (vSer), the relationship between devices fell along the line of unity and had an R^2^ = 0.94, and had the narrowest confidence intervals (0.91–1.10) (Figure 4).

#### 3.1.4. Multiple Regression Analysis of All *IronScan*™ Comparisons to Immulite 2000 Xpi

To test for the combined contributions of the type of blood sampling (comparison B) and the medium of blood analyses (comparison C) to the relationship of the prescribed analysis (comparison A), we performed a multiple regression analysis on a subset of the sample (*n* = 35; Figure 1) that had ferritin measured across devices and all blood sampling methods (comparisons A, B, and C), with Immulite sVer as the dependent variable. The R^2^ = 0.85 was estimated for the bivariate comparison of *IronScan™* cWB versus Immulite vSer, while the addition of *IronScan™* vSer raises the R^2^ by 10% to 0.95. Lastly, the addition of *IronScan™* vWB increases the R^2^ above *IronScan™* vSer alone by 6% to 0.91.

#### 3.1.5. Identification of Iron Deficiency Using the WHO or Clinical Cutoffs

The clinical purpose of blood ferritin measurements is to assess iron stores in the diagnosis of iron deficiency or iron deficiency anemia. Depending upon the cutoff (clinical or WHO), 22–46% of volunteers were classified as iron deficient using the gold standard, Immulite vSer (Table 3; Supplemental Table S1). At the clinical cutoff of 12 ng/mL, the *IronScan*™ sensitivity and specificity were 33% and 100%, respectively. At the WHO cutoff of 30 ng/mL, the *IronScan*™ sensitivity increased to 85%, and specificity remained high at 96%. 

## 4. Discussion

Iron deficiency affects nearly 30% of the world’s population [8], and the WHO recommends ferritin measurements for diagnostic and screening purposes as well as to assess and monitor the impact of iron-related interventions [9]. Since venipuncture is required to obtain ~5 mL of serum, a phlebotomist is needed for venous serum (vSer) ferritin determinations. Venipuncture samples must be processed and typically transported to a clinical laboratory using specific labeling and handling requirements, i.e., a continuous cold chain, biological hazard with a regulatory-approved, bench-top ferritin analytical device. The process could be simplified with point-of-care devices capable of measuring ferritin in a very small sample of capillary blood. Furthermore, the economic impact of a point-of-care device would reduce patient costs. As a standard lateral flow assay, the *IronScan™* system is estimated to sell at a commercial level for approximately USD 100 and at USD 1 per test.

This study validates *IronScan™* as a point-of-care diagnostic device for measuring ferritin concentrations in whole blood and serum. *IronScan™* has a wide linear range, with values outside this range reported as <5 or >150 ng/mL [15]. We established the utility and validity of using *IronScan™* with cWB (finger stick) to diagnose iron deficiency (Table 3) with a small sample size represented mostly by women of reproductive age who are at greater risk for iron deficiency. Fewer than 5% of samples fall outside the 95% confidence interval (Figure 2B) compared to the gold standard method (Immulite 2000 Xpi), and these outliers were observed with cWB ferritin concentrations >60 ng/mL (Figure 2B), i.e., ferritin concentrations well outside the upper range for diagnosing iron deficiency in which false positives would be observed. The disparity between ferritin values in capillary versus venous blood samples (Table 2) is known, constant, and linear, within the range of 0 to 200 ng/mL [20]. The correlation between ferritin measurements remained high (R^2^ = 0.86) when venous blood was analyzed with both diagnostic devices (t 3), providing evidence that when a phlebotomist collects blood samples by venipuncture, *IronScan™* could be used onsite to omit the transportation of biological samples under refrigeration to a remote laboratory for ferritin analysis. A high correlation (R^2^ = 0.94) along the line of unity was observed when venous blood provided serum for both devices (Figure 4). Since centrifuging whole blood to obtain serum is relatively straightforward, onsite use of *IronScan™* to measure ferritin concentrations in venous serum samples would eliminate the need to transport serum samples to a remote clinical laboratory while still requiring trained personnel to take human blood. Furthermore, the identification of iron deficiency with *IronScan™* using the WHO cutoff of 30 ng/mL yielded high sensitivity and specificity (Table 3); the sensitivity increased with the higher cutoff without compromising specificity, suggesting low misclassification of iron deficiency. The sensitivity of identifying iron deficiency using the clinical cutoff of 12 ng/mL was low (Table 2); a larger sample size may improve the sensitivity of *IronScan™* using the clinical cutoff. There are many applications for a point-of-care device capable of measuring ferritin concentrations. Maternal iron status in pregnancy is associated with the iron status of the child at birth and to a lesser extent with the child’s neurodevelopment [21]. Routine Hb measurement is generally recommended at each trimester of pregnancy, but hemodilution is a confounder [22]. A ferritin test more accurately predicts iron status; however, a retrospective cohort population-based study in Ontario, Canada found that only <59.4% of pregnant women had their ferritin levels measured, and that the odds of a ferritin test being administered were negatively associated with annual household income [11]. Socioeconomic factors should not be a barrier to iron screening. 

With respect to the screening of children 6–18 months of age, it is predicted that both universal and targeted (negative ferritin, Hb and CRP) screening programs in Ontario, Canada would be more cost-effective than no screening, based on willingness-to-pay thresholds of $50,000 and $100,000, per QALY [13]. Estimates of direct and indirect costs included laboratory costs (services, administration, specimen collection fees) and parent costs (salary loss while taking child for testing and travel expenses), which would be eliminated or reduced with point-of-care testing.

Among blood donors, female sex and intensity of screening are major risk factors for iron deficiency [23,24,25,26]. Ferritin screening of blood donors could help maintain adequacy of blood supply by reducing hemoglobin (Hb) deferrals that result in lower return rates and donation frequency [27]. In short, point-of-care testing could help reduce false negatives in screening and help maintain blood banks.

## 5. Conclusions

Ferritin measurements from cWB, vWB, and vSer using *IronScan™* when ferritin concentrations are <150 ng/mL are linearly correlated with low misclassification of iron deficiency relative to using ‘approved’ venous blood samples (vSer) and a lab-based diagnostic device (Immulite 2000 Xpi). We conclude that (1) blood ferritin determinations below 150 ng/mL and the risk of a false iron deficiency diagnosis when ferritin < 30 ng/mL are similar between Immulite and *IronScan™*, and (2) *IronScan™* is a reliable and accurate point-of-care diagnostic system with practical field or point-of-care applications in human subjects.

## Figures and Tables

**Table 1:** Subject characteristics (*n* = 44).

	Mean ± SD	Range
Age, year	33.8 ± 11.0	19–61
Height, cm	168.5 ± 7.8	150–178
Weight, kg	70.1 ± 15.3	52–106
BMI, kg/m^2^	24.7 ± 4.6	19–41

**Table 2:** Ferritin values and ranges across devices and blood sampling methods ^1^.

	Mean ± SD	Range
Ferritin from Immulite vSer, ng/mL	41.1 ± 30.7	5.4–119.0
Ferritin from *IronScan™* cWB, ng/mL	40.7 ± 27.3	7.6–114.0
Ferritin from *IronScan™* vWB, ng/mL	56.3 ± 40.1	5.0–154.0
Ferritin from *IronScan™* vSer, ng/mL	46.1 ± 32.5	7.2–137.6

^1^ Blood samples were collected between 27–29 June 2022. vSer, venous serum; cWB, capillary whole blood; vWB, venous whole blood.

**Table 3:** Identification of iron deficiency (*n* = 44) ^1^.

	Sensitivity (%)	Specificity (%)
≤12 ng/mL (clinical)	33	100
≤30 ng/mL (WHO)	85	96

^1^ Sensitivity is the probability that the *IronScan™* will diagnose iron deficiency among those with iron deficiency as measured by the Immulite 2000 Xpi (true positives). Specificity is the fraction of those without iron deficiency (true negatives as measured by the Immulite) that test as non-iron deficient on the *IronScan™*.

## Data Availability

Data described in the manuscript are held with the Division of Nutritional Sciences at Cornell University and will be made available upon request pending approval by the corresponding author.

## Supplementary Materials

The following supporting information can be downloaded at: https://www.mdpi.com/article/10.3390/nu15061305/s1, Table S1: Ferritin values used for sensitivity and specificity analyses.
