# Supplementary material for: Using Capillary Whole Blood to Quantitatively Measure Ferritin: A Validation Trial of a Point-of-Care System
Source: Nutrients. 2023 Mar 7;15(6):1305. doi: 10.3390/nu15061305 (PMC10052100; doi:10.3390/nu15061305)
Supplement: Supplementary file 1 [file nutrients-15-01305-s001.zip › nutrients-2252804-supplementary.pdf]

Subject #	Gender	Immulite vSer	*IronScan ™* cWB
1	F	25.3	31.0
2	M	88.0	75.0
3	F	11.6	16.0
4	F	17.5	28.0
5	M	8.4	20.0
6	F	10.8	14.0
7	F	22.3	19.0
8	F	42.9	49.0
9	F	11.2	25.0
10	M	59.3	71.0
11	F	46.8	73.8
12	F	48.0	38.8
13	F	53.1	62.9
14	F	27.3	22.2
15	F	89.1	87.8
16	F	8.7	7.6
17	F	117.7	114.0
18	F	11.9	35.0
19	F	5.4	12.8
20	F	73.9	61.3
21	M	119.0	113.0
22	F	16.7	30.4
23	F	43.8	48.9
24	M	102.8	91.1
25	F	41.6	32.5
26	F	22.4	15.6
27	F	11.4	27.6
28	F	32.3	24.9
29	F	33.7	31.3
30	F	19.5	19.5
31	F	92.4	57.2
32	M	65.8	46.1
33	F	6.9	8.6
34	F	24.6	20.2
35	F	35.8	33.3
36	F	35.6	30.4
37	F	45.7	60.1
38	F	38.9	37.1
39	F	44.8	49.4
40	F	60.6	49.6
41	M	74.9	86.0
42	F	12.0	9.6
43	F	14.8	24.2

44	F	32.4	39.6
